# Urban tolerance is phylogenetically constrained and mediated by pre‐adaptations in African bats

**DOI:** 10.1002/ece3.9840

**Published:** 2023-03-08

**Authors:** Genevieve E. Marsden, Dalene Vosloo, M. Corrie Schoeman

**Affiliations:** ^1^ Centre for Functional Biodiversity, School of Life Sciences University of KwaZulu‐Natal Durban South Africa; ^2^ School of Life Sciences University of KwaZulu‐Natal Durban South Africa

**Keywords:** Chiroptera, phylogenetic signal, pre‐adaptation, urban

## Abstract

With increasing urbanization, particularly in developing countries, it is important to understand how local biota will respond to such landscape changes. Bats comprise one of the most diverse groups of mammals in urban areas, and many species are threatened by habitat destruction and land use change. Yet, in Africa, the response of bats to urban areas is relatively understudied. Therefore, we collated data on urban presence, phylogenetic relationship, and ecological traits of 54 insectivorous bats in Africa from available literature to test if their response to urbanization was phylogenetically and/or ecologically driven. Ancestral state reconstruction of urban tolerance, defined by functional group and presence observed in urban areas, suggests that ancestral African bat species could adapt to urban landscapes, and significant phylogenetic signal for urban tolerance indicates that this ability is evolutionarily conserved and mediated by pre‐adaptations. Specifically, traits of high wing loading and aspect ratio, and flexible roosting strategies, enable occupancy of urban areas. Therefore, our results identify the traits that predict which bat species will likely occur in urban areas, and which vulnerable bat clades conservation efforts should focus on to reduce loss of both functional and phylogenetic diversity in Africa. We, additionally, highlight several gaps in research that should be investigated in future studies to provide better monitoring of the impact urbanization will have on African bats.

## Graphical abstract

African insectivorous bats (order Chiroptera) can be classified into urban avoiders, adapters, or exploiters based on wing morphology and roost specificity. These traits and a species' phylogenetic history drive pre‐adaptations for inhabiting urban areas. The ancestor of these African species likely had traits aligning with a modern urban adapter bat.

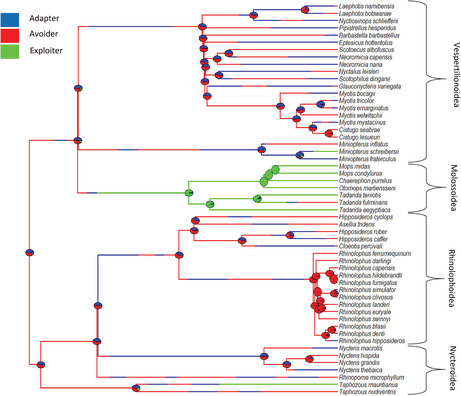

## 1 INTRODUCTION

Urbanization is expanding rapidly worldwide and threatening biodiversity. In 2020, 56% of the world's population (4.4 billion people) lived in urban areas, with this number projected to increase to 68% (over 6 billion people) by 2050 (The World Bank, 2022; United Nations, 2018). Accordingly, urban areas will have to expand rapidly to accommodate these numbers, with much of this projected expansion occurring in Africa (United Nations, 2018). In urban areas, biodiversity faces many challenges such as habitat loss, fragmentation, human–wildlife conflicts, and pollution (Ditchkoff et al., 2006). Species in urban areas suffer fatalities such as road‐kill (Kent et al., 2021), eradication as pests, exposure to chemicals and other pollutants (Ditchkoff et al., 2006), and predation by introduced species such as domestic cats (Marzluff & Ewing, 2008). On the other hand, some species thrive in urban landscapes. For instance, scavengers benefit from the build‐up of garbage (O'Connor, 1993), opportunistic insectivorous bats hunt large and predictable swarms of insects near urban waterbodies and street lights (Naidoo et al., 2011; Schoeman, 2016; Stone et al., 2015), and some large mammals find shelter and refuge from natural predators in urban structures (Bateman & Fleming, 2012; Marzluff & Ewing, 2008). Thus, urbanization can be beneficial for certain taxa and maintain, or even increase, biodiversity (Lee et al., 2021; McKinney, 2008). Understanding the way individual species respond to urban areas is important for sustainable urban development and conservation of resident species, particularly in biodiversity‐rich regions.

Bats (order Chiroptera) are often overlooked as part of urban wildlife (Voigt & Kingston, 2016). However, bats make up a fifth of all mammal species and are often the most diverse mammal group in urban areas (Jung & Threlfall, 2016). Insectivorous bats are ecologically important worldwide, particularly in the control of disease and insect pests (Kunz et al., 2011). However, many species are threatened (IUCN, 2021; Racey, 2009), with urbanization causing habitat destruction and fragmentation that is detrimental to bat populations (Mickleburgh et al., 2002). While some bat species largely avoid urban areas, other species are abundant and take advantage of the novel foraging and roosting sites in urban areas (Avila‐Flores & Fenton, 2005; Jung & Threlfall, 2016; Schoeman, 2016). Based on their response to urban areas, insectivorous bats can be classified into three groups: urban exploiters, urban adapters, or urban avoiders (Jung & Kalko, 2011; McKinney, 2002). Urban exploiters are species that are almost dependent on urban resources and can become abundant in urban areas, urban adapters are common in suburban areas and readily use urban resources but are not reliant on them, and urban avoiders hardly occur in urban areas, unable to use urban resources (McKinney, 2002). In bats, these classifications often depend on the functional grouping of species and their use of urban resources (Avila‐Flores & Fenton, 2005; Jung & Kalko, 2011).

Insectivorous bats are divided into three functional groups: open‐air, narrow‐edge, and narrow‐space bats (Denzinger & Schnitzler, 2013). Species are adapted to each of these environments via specialized wing morphology and echolocation for locomotion as well as optimal prey detection and capture (Aldridge & Rautenbach, 1987; Schnitzler & Kalko, 2001). Open‐air foragers have wings with high aspect ratios, high wing loading, and pointed wing tips that enable fast flight over long distances, and echolocation with low frequencies and long duration that are optimal to detect prey in open spaces without background clutter (Denzinger & Schnitzler, 2013). Narrow‐edge space foragers have intermediate aspect ratios and wing loading with rounded tips which favor flexible foraging at the edge of vegetation and open spaces. Their echolocation characteristics enable narrow‐edge space bats to detect prey in the vicinity of clutter, but where there is enough space that prey and background signals do not overlap (Denzinger & Schnitzler, 2013). The wings of narrow space foragers have low aspect ratios and wing loading with very rounded tips enabling agile flight in narrow spaces, with echolocation well adapted to detect echoes of insects against the cluttered background's interference (Denzinger & Schnitzler, 2013). Urban landscapes tend to benefit open‐air bats because ephemeral food resources and roosts are spread across wide, open spaces (Avila‐Flores & Fenton, 2005; Jung & Threlfall, 2018). Moreover, in urban areas, roosts on roofs of houses and buildings, and crevices in buildings are readily utilized by bats that have flexible roosting requirements, whereas cave reliant bat species are typically excluded (Bergeson et al., 2015; Jung & Threlfall, 2016; Schoeman, 2016). Thus, in combination, functional traits and roosting ecology of insectivorous bats may determine their tolerance of urbanization (Jung & Threlfall, 2018).

Bat families generally have distinct functional traits, and hence, likelihood of presence in urban habitats (Denzinger & Schnitzler, 2013; Jung & Threlfall, 2016). For instance, Molossidae, the free‐tailed bats, are open‐air foragers with flexible roost preferences in crevices, tombs, and houses, whereas Rhinolophidae, the horseshoe bats, are narrow space bats that are obligate cave roosters (Denzinger & Schnitzler, 2013). Consequently, Molossidae are frequently found foraging and roosting in urban areas (Avila‐Flores & Fenton, 2005), whereas Rhinolophidae are often conspicuously absent (Jung & Threlfall, 2018; Schoeman, 2016; Schoeman & Waddington, 2011). Although many family groups generally fit into one of these functional groups (see Denzinger & Schnitzler, 2013), some families such as Vespertilionidae are more variable, with different species belonging to various functional groups (Jung & Threlfall, 2016; Monadjem et al., 2020). Therefore, responses to urbanization may be underpinned by phylogenetic history (measured by phylogenetic signal) where closely related species are significantly more likely to respond similarly to urban areas than distantly related species (Blomberg & Garland, 2002). Jung and Threlfall (2018) suggested that phylogenetic relationships may play some role in urban tolerance of bats but indicated the need for further studies to confirm this. Phylogenetic conservatism may hinder evolutionary adaptations that enhance the ability of species to utilize resources in urban habitats (Ackerly, 2009). Pre‐adaptations, traits evolved to previous conditions that serve as an advantage in the novel environment (Blomberg & Garland, 2002), often mediate the successful invasion into novel environments (Bock, 1959), and subsequent rapid adaptive evolution allows persistence in the new environment (Jenkins & Keller, 2011; Sultan et al., 2012; Whitney & Gabler, 2008). How these processes contribute to urban success of bats has rarely been studied, yet is key to predicting extinction risks and formulating effective conservation measures.

In this light, we asked what role evolutionary history played in current patterns of urban tolerance of African insectivorous bats. Africa is a fast‐developing continent where 20% of its 320 bat species are listed as threatened (ACR., 2018; IUCN, 2021; United Nations, 2014), yet it is markedly understudied compared to other continents (Collins et al., 2021; Magle et al., 2012). We tested the phylogenetic signal of urban tolerance (in terms of urban avoider, adapter, or exploiter status) in African bat species, and reconstructed the ancestral state of urban tolerance. If successful urban exploiters were pre‐adapted for urban areas, we predicted significant phylogenetic signal in urban tolerance, with the reconstructed ancestral node in the urban exploiter state. We also tested for evidence of co‐evolution between urban presence and the functional traits and roosting ecology of bat species. Previous studies found that high wing aspect ratio, low peak echolocation frequency, and high roost specificity were important traits in urban exploiters in other regions (Jung & Threlfall, 2018; Wolf et al., 2022). Thus, we predicted significant correlations between urban presence and echolocation, wing morphology, and roost specificity for African species.

## 2 METHODS

### 2.1 Data collection

We compiled a list of all mainland African insectivorous bat species using ACR (2018), Kingdon (2013), and Monadjem et al. (2020). We collected aspect ratio, wing loading, peak echolocation frequency, roost ecology, and functional group data for each of these species from these sources and other available literature (ACR., 2018; Aldridge & Rautenbach, 1987; Kingdon, 2013; Monadjem et al., 2020; Norberg & Rayner, 1987; Salsamendi et al., 2005). Available ecological trait data and presence in the phylogenetic super‐tree (Jones et al., 2005) reduced our data set from an initial 219 species to 54 species for statistical analyses [data available: https://doi.org/10.5061/dryad.k3j9kd5b9]. We then determined whether these species were present in urban (including suburban or peri‐urban) areas within their range (personal communication P. Webala, I. Tanshi and M.C. Schoeman; and Ancillotto et al., 2015; Andreani et al., 2019; Dekker et al., 2013; Fenton et al., 2002; Geldenhuys et al., 2013; Hoye & Spence, 2004; Jacobs & Barclay, 2009; Kurek et al., 2020; Lane et al., 2022; Legakis et al., 2000; O'Malley et al., 2020; Roswag et al., 2019; Schoeman, 2016; Schoeman & Waddington, 2011; Taylor et al., 1999; Wojtaszyn et al., 2013) and recorded this information as presence (1) or absence (0) in urban areas. We categorized roost specificity for each species as: utilizing 1 roost type = high, 2 roost types = medium, and ≥3 roost types = low. Each of the following was considered a different roost “type”: caves and mines, tree crevices (behind bark and tree holes), foliage, rock crevices, exposed outer walls of houses/buildings, roofs of houses/buildings, and road culverts. Roost specificity was classified regardless of the surrounding habitat type (e.g., open vs. narrow space) or landscape (e.g., highly urbanized vs. more rural areas) where the roost was found.

We categorized bats into urban exploiters, adapters, or avoiders after Jung and Kalko (2011) and Schoeman (2016) based on wing morphology and roost habits. Urban exploiters are open‐air bats with high wing loading and aspect ratios and highly flexible roost habits that readily use anthropogenic resources; urban adapters are narrow‐edge space bats with intermediate wing loading and aspect ratios, and fairly flexible roosting habits; and urban avoiders are narrow‐space bats with restricted roosting requirements, such as obligate cave roosters (Jung & Kalko, 2011; Schoeman, 2016).

### 2.2 Phylogenetic analyses

We used the super‐tree by Jones et al. (2005) and pruned it to 54 bat species for which we had ecological data in the geiger (Harmon et al., 2008) and ape (Paradis & Schliep, 2019) packages of R statistical software version 4.1.0 (R Core Team, 2021). We used the “fix.poly” function in RRphylo (Castiglione et al., 2021) to resolve polytomies of this tree for all subsequent phylogenetic analyses.

To test phylogenetic signals and reconstruct ancestral states, the model of evolution for the trait in question must be known. Therefore, we first determined the model of evolution of urban tolerance among states of “urban exploiter,” “urban avoider,” and “urban adapter” in the pruned phylogeny, using the “fitDiscrete” function in the geiger package. We compared the fit of the three models of evolution for urban tolerance using weighted Akaike's information criterion (AIC). Discrete characters can evolve under three models of evolution that govern the rate at which a trait is likely to evolve along the branches of the tree: equal rates (ER; the trait evolves at a uniform rate across the tree regardless of which states it is changing between), all‐rates‐different (ARD; the trait evolves at different rates across the tree regardless of which states it is changing between), and symmetric models (SYM; the rate of evolution varies across the tree but the rate of change between two states is symmetrical in that the forward and backward rates of evolution between those two particular states are equal). The best fitting model based on weighted AIC comparison was then used as the model of evolution to test phylogenetic signal and reconstruct ancestral states.

We measured the degree of phylogenetic signal using the same function “fitDiscrete” in geiger, with the tree transformation of Pagel's lambda (*λ*). This provides a value for Pagel's *λ* between 1 and 0, where 1 = strong phylogenetic signal and 0 = no phylogenetic signal. Included in the output is the estimated AIC value for the tree. We tested the fit of this lambda value against a lambda value of 0 for urban tolerance evolution on the tree by creating a tree of lambda = 0 and comparing the weighted AIC values calculated in each. We reconstructed the ancestral state of urban tolerance with stochastic character mapping of the joint posterior probabilities of the internal nodes of the tree (Bollback, 2006; Huelsenbeck et al., 2003) using the packages phytools (Revell, 2012) and ape. We ran 1000 simulations and plotted the average probabilities for each node in any given state as a pie chart at each node onto the phylogenetic tree.

Finally, we tested which traits – echolocation, wing loading, aspect ratio, or roost specificity – significantly predicted the presence/absence of bat species in urban areas. Roost specificity was coded as dummy variables. We fit the phylogenetic generalized linear model (PGLM) developed by Ives and Garland (2010) using the package Phylolm (Ho & Ané, 2014) in R. PGLM takes the strength of the phylogenetic correlation in the binary dependent variable into account to calculate the regression coefficients of the independent variables for both continuous and discrete multistate traits. The output provides regression coefficient estimates with their standard errors, Wald's *Z*‐values, and associated *p*‐values. Parametric bootstrapping provided confidence intervals for the estimates. We used an alpha value of .05 to determine significance of parameters. The model phylogenetic signal (*α* = .02) was low. A standard GLM run in the R base package yielded slightly different results, indicating that the phylogeny affects result even at this low *α*. We, therefore, report results of the PGLM. We also estimated the phylogenetic signal of the dependent variable using the same function (without independent variables) as well as the delta estimate (Fritz & Purvis, 2010) using “phylo.d” in the caper package (Orme, 2012). We estimated the phylogenetic signal of the continuous traits with “phylo.sig” in Phytools and the phylogenetic signal of roost specificity (categorical data) with “fitDiscrete” as above.

## 3 RESULTS

Among 54 bat species, there were 30 absent (55%) and 24 present in urban areas; 23 (43%) were classified as urban avoiders, 19 (35%) as adapters and 12 (22%) as exploiters.

**FIGURE 1:**
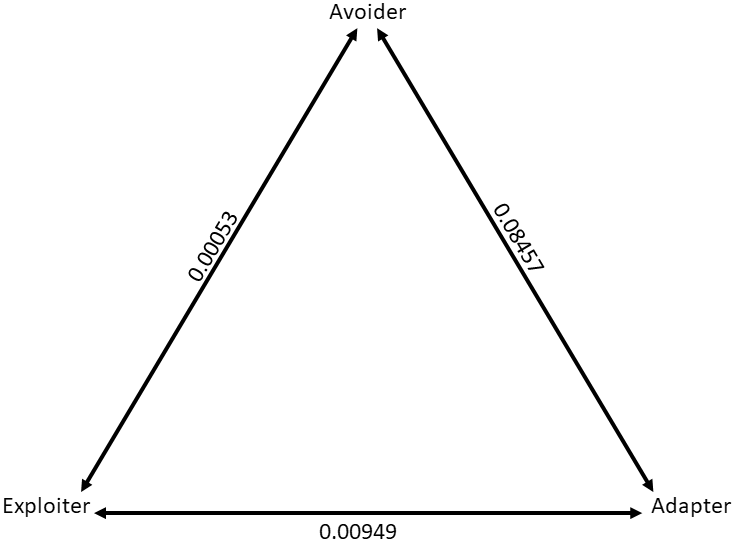
Evolutionary changes between states of urban tolerance represented by the “symmetrical” model (SYM). Transitions among states (avoider, adapter, or exploiter) are shown for the trait “urban tolerance.” Transitions are represented by double‐ended arrows (change can occur in either direction between states). Values indicate the rates of change between each pair of states, under a model where the rate between each pair is allowed to be different, but the rate of switching forward or backward between pairs of states is equal. The weighted AIC value for this model = 84% support compared to “equal rates” (2%) and “all‐rates different” (14%) models.

Urban tolerance in these African bat species evolved under a “symmetrical” evolutionary model. Here, the rates of change between two states of urban tolerance are not constrained to be equal to the rate of change between any other two states of urban tolerance, but the reverse (forward or backward) change between the same two states is equal (Figure 1). All transition rates were low, but the highest rate of switching occurred between urban avoider and urban adapter states implying transitions between these two states were most common within the phylogeny (Figure 1).

**FIGURE 2:**
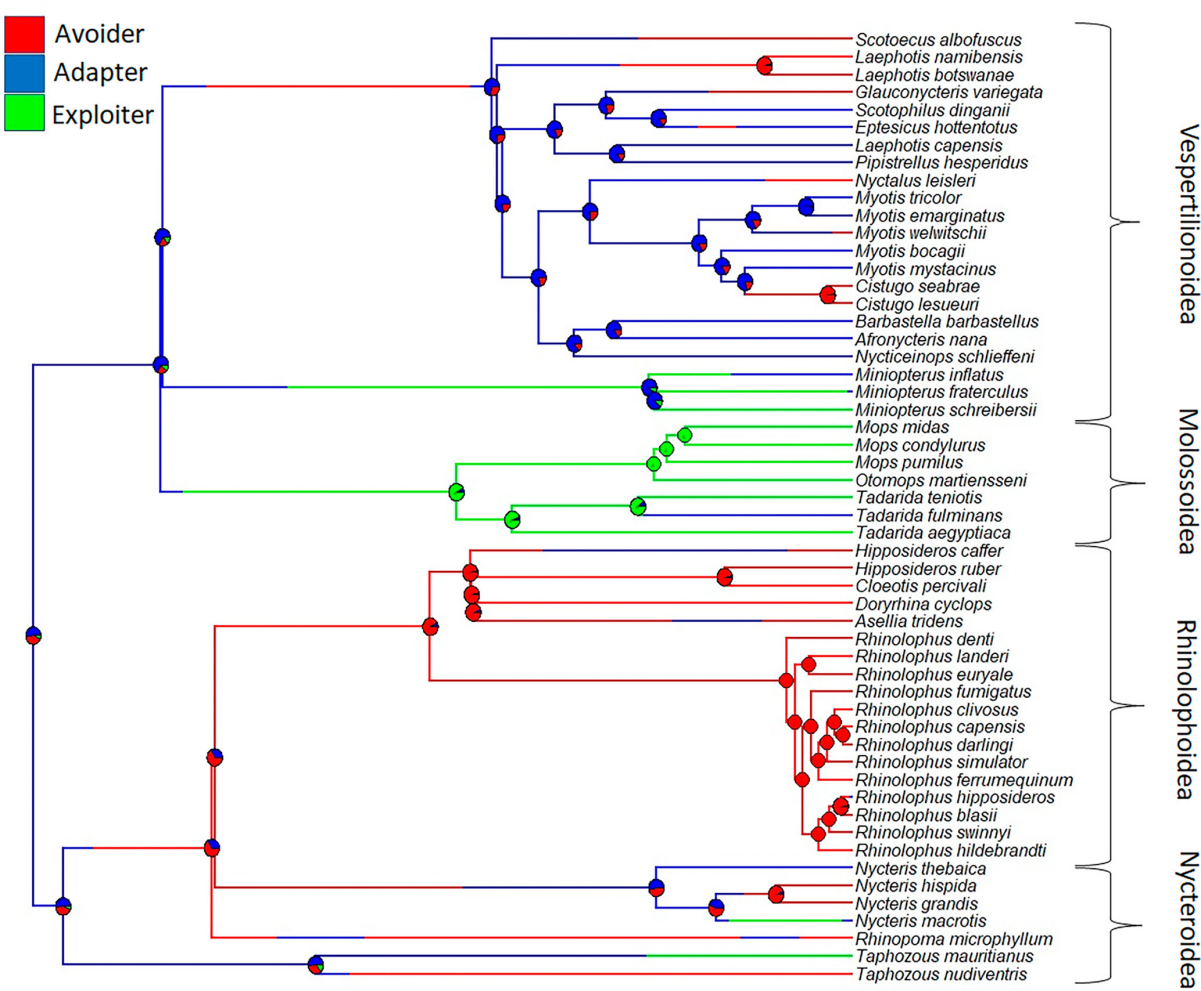
Phylogenetic tree of African bats with known states of urban tolerance at the tips and calculated posterior probabilities for states of internal nodes. The lambda value for urban tolerance on this tree = 0.78. The probabilities of the ancestral node in each state = 0.48 for urban adapter, 0.45 for urban avoider, and 0.07 for urban exploiter. States of urban tolerance are color coded (urban exploiter = green, urban adapter = blue, and urban avoider = red). Superfamily groups are indicated on the right (after ACR, 2018).

Pagel's *λ* for urban tolerance = 0.78, indicating that there is a significant phylogenetic signal in the manner urban tolerance is distributed across African bat species, further supported by the significant AIC value of 0.99 for this model. The reconstructed ancestral state of urban tolerance was 48% likely “urban adapter” (root node state: urban adapter = 0.48, urban avoider = 0.45, and urban exploiter = 0.07 [Figure 2]). The “urban avoider” state was also ancestral, whereas “urban exploiter” is the most derived state, evolving once early in Molossidae and, more recently, once in Vespertilionidae and once in Emballonuridae (Figure 2).

The PGLM of urban presence among African bat species showed that wing loading, aspect ratio, and roost specificity significantly predicted urban presence (Table 1). High wing aspect ratio significantly increases the chance of species presence in urban areas (*β* = 1.02 ± 0.45, *Z* = 2.25, *p* = .025). Similarly, low wing loading significantly decreases the chance of species presence in urban areas (*β* = −0.42 ± 0.15, *Z* = −2.78, *p* = .005) (Table 1). High roost specificity significantly decreases the chance of species presence in urban areas (*β* = −2.01 ± 0.87, *Z* = −2.30, *p* = .021) (Table 1). Aspect ratio, wing loading, and echolocation also displayed strong phylogenetic signals within Chiroptera (*λ* = 0.97, *λ* = 0.98, and *λ* = 0.83 respectively), however, roost specificity and urban presence did not (*λ* = 0.18 and *λ* = 0.34, respectively; Table 2).

**TABLE 1:** Regression coefficient estimates with associated standard errors and significances for the phylogenetic generalized linear model “Urban presence ~ wing loading + aspect ratio + echolocation + roost specificity.”

	Estimate (*β*)	Standard error	Lower CI	Upper CI	*Z*‐score	*p*‐Value
Intercept	−1.25	2.86	−1.38	−1.00	−0.44	.662
Aspect ratio	1.02	0.45	0.54	1.51	2.25	.025*
Wing loading	−0.42	0.15	−0.70	−0.17	−2.78	.005*
Echolocation	−0.02	0.01	−0.04	−0.01	−1.69	.091
Medium roost specificity	−0.56	0.72	−0.98	0.24	−0.78	.434
High roost specificity	−2.01	0.87	−2.48	−1.41	−2.30	.021*

*Note*: Confidence intervals were calculated by parametric bootstrapping based on the model *α* = .02. Significant parameter estimates are indicated with asterisks (*).

**TABLE 2:** Phylogenetic signal of the functional traits, roost specificity, urban presence, and urban tolerance classifications.

	Pagel's *λ*	Delta (*δ*)
Urban presence	0.34	0.38
Aspect ratio	0.97	
Wing loading	0.98	
Echolocation	0.83	
Roost specificity	0.18	
Urban tolerance	0.78	

*Note*: Pagel's *λ* was calculated for categorical and continuous data, and the delta variable was additionally calculated for the binary variable.

## 4 DISCUSSION

This study is the first to investigate the evolutionary drivers of urban tolerance in African bats. We found significant phylogenetic signal in urban tolerance among insectivorous African bat species, and the ancestral state aligned with both urban adapters and urban avoiders. Therefore, the ancestral bat of these African species was likely a narrow‐edge space forager with traits to successfully utilize urban landscape features if they had existed. Urban adapter and exploiter states evolved prior to urbanization, although exploiters evolved much later than adapters in the phylogeny – specifically in the Molossidae family which diverged relatively recently from the other families in this phylogeny. Thus, states indicative of success in urban areas are driven by factors that pre‐date urbanization, but extant bats did not evolve from an ancestral species that possessed characteristics to exploit urban areas. Transition rates between different states of urban tolerance in bats were low, indicating infrequent state changes and trait conservatism within the phylogeny (Bell et al., 2017). These low rates are probably the reason for the significant phylogenetic signal for urban tolerance among extant bat species. These results indicate that bat species are most likely to inhabit environments they are well suited to rather than undergo rapid adaptive evolution (Ackerly, 2009). Therefore, tolerance of urban areas is mediated by pre‐adaptations that evolved in non‐urban environmental conditions and were present in the common ancestor of these bats (Ackerly, 2009; Blomberg & Garland, 2002). Similarly, in birds, urban tolerance is characterized by a suite of preadapted traits – such as short flight distances (Møller, 2009) and high‐frequency songs (Hu & Cardoso, 2009) – and urban exploiters are mostly from particular clades (Sol et al., 2017). These results suggest that increased urbanization spread may be linked to marked loss of phylogenetic diversity in local assemblages (Callaghan et al., 2021; Sol et al., 2017).

On the other hand, we found that the urban exploiter state recently appeared in two clades – Emballonuridae and Vespertilionidae. Generally, long lifespans and generation times, like those of bats, decrease adaptation rates (Jones et al., 2003). However, it is possible that in established urban populations, rapid evolution can work in tandem with pre‐adaptations to promote persistence of these populations (Jenkins & Keller, 2011; Yeh, 2004). Moreover, strong novel selection pressure may act on mechanisms of phenotypic or behavioral plasticity such that populations rapidly shift the way they use resources in the environment, without genotypic or evolutionary change (Charmantier et al., 2008; Garland & Kelly, 2006). For example, some urban fruit bat populations have adjusted their diets (Egert‐Berg et al., 2021), and some urban birds can alter their song frequency (Slabbekoorn & den Boer‐Visser, 2006) in response to noise in urban areas. In insectivorous bats, echolocation peak frequency and bandwidth may display plasticity as bats can adjust these to prevent masking from acoustic interference altitudinally, geographically, and in response to some anthropogenic noises (Bunkley et al., 2015; Gillam et al., 2009; Jiang et al., 2015). Thus, the role of adaptive phenotypic plasticity in insectivorous bats should be further investigated as an avenue of adapting to urbanization.

In support of our predictions, wing morphology and roost specificity best predicted the presence of bats in urban areas. Specifically, bats pre‐adapted for urban areas have high wing loading and aspect ratio, and low‐to‐medium roost specificity. Bats with intermediate‐to‐high wing loading and aspect ratios are highly mobile, with good dispersal abilities and moderate‐to‐fast flight speeds (Arita & Fenton, 1997; Denzinger & Schnitzler, 2013; Norberg & Rayner, 1987). These traits are beneficial in urban environments because resources are distributed patchily across the landscape (Jung & Kalko, 2011; Jung & Threlfall, 2018; Piano et al., 2017). Moreover, in urban areas, artificial night lighting is ubiquitous, and provides an important source of concentrated insect prey for narrow‐edge space and open‐air species (Gaisler et al., 2006; Schoeman, 2016; Tomassini et al., 2014), whereas slow‐flying bats with low aspect ratio and wing loadings avoid lit areas and instead rely on vegetated habitats (Hourigan et al., 2006; Jung & Kalko, 2010; Rydell, 1992). These traits also display strong phylogenetic signals and therefore, allow conclusions on species responses to urban areas based on evolutionary history. Our results support those of a global meta‐analysis (Jung & Threlfall, 2018) that found high aspect ratio and flexible roosting strategies promote urban tolerance. Although we found that high wing loading was also a significant driver of urban tolerance, the global analysis included few African species (Jung & Threlfall, 2018). Similarly, Wolf et al. (2022) suggest that flexible roosting strategies were important for urban tolerance, in addition to low echolocation peak frequency and broad bandwidth duration. Overall, it appears that high mobility and flexible roost habits are the most important predictors of urban tolerance and can be used to determine species‐specific responses to urban areas for bats (Jung & Kalko, 2011; Jung & Threlfall, 2018).

Some African bat species with traits that favor wide dispersal, such as the open‐air *Nyctalus* species, *Tadarida fulminans*, and *Taphozous nudiventris*, were absent from urban environments. Although this may be due to the lack of records of these species in African urban areas, roost specificity (e.g., *T. fulminans*) or dietary requirements may prevent species from occupying urban areas (Jung & Threlfall, 2018; Palacio, 2019). Roosts are crucial resources for bats, and often limiting in natural habitats (Mickleburgh et al., 2002; Zukal et al., 2017). Urban areas provide various roost types including roofs of houses, crevices in the walls of buildings, attics, and the eaves of houses (Monadjem et al., 2020; Russo & Ancillotto, 2015; Voigt & Kingston, 2016). Bat species that select roosts in buildings over natural roosts gain significant reproductive benefits and protection from predation (Fuentes‐Montemayor et al., 2014; Johnson et al., 2019; Lausen & Barclay, 2006; O'Malley et al., 2020; Voigt et al., 2016). However, obligate cave roosting bats and other species with specific roosting habits are unlikely to find suitable roosts in urban areas (Russo & Ancillotto, 2015). This corroborates previous findings that roosting ecology determines the presence of bat species in urban areas (Duchamp et al., 2004; Jung & Threlfall, 2018; Wolf et al., 2022). Furthermore, roost specificity exhibited little phylogenetic signal, and its importance in determining which species inhabit urban areas may explain why urban presence had relatively low phylogenetic signal. Because relatively little is known about the roosting or dietary ecology of many African bat species (Monadjem et al., 2020), more research on roost and diet requirements is an important step to identify species vulnerable to urbanization.

Although more than 50% of bat species in this study appeared to be sensitive to urbanization, only 12 were classified as urban exploiters. Molossidae were almost all urban exploiters, but Rhinolophoidea species were phylogenetically constrained to, almost exclusively, avoid urban landscapes. This closely aligns with locomotion and roosting ecology – Molossidae are adapted for open space, with flexible roosting strategies, whereas Rhinolophoidea are narrow space bats with low wing loading and aspect ratios, high echolocation frequencies, and are mainly obligate cave roosters. The only Molossidae species absent from urban areas (*T. fulminans*) has very high wing loading coupled with high roost specificity (Monadjem et al., 2020). Vespertilionidae and Miniopteridae species are predominantly urban adapters, with urban avoiders and exploiters more derived in several genera. Notably, urban avoidance is highly prevalent in *Cistugo* and *Laephotis* genera and can probably be attributed to roost specificity and the rarity of these species (Monadjem et al., 2020). On the other hand, *Nycticeinops schlieffenii* possesses no obvious advantageous traits for urban areas yet can inhabit cities (Hoye & Spence, 2004). The emballonurid *T. nudiventris* is a widely distributed open‐air bat with medium‐to‐high wing loading and flexible roost requirements, yet there is no evidence that this species is an urban resident, suggesting that advantageous pre‐adaptations are not a guarantee for success in urban landscapes (Moiron et al., 2015). Alternatively, available observational data may be limited or incomplete – urbanization may not have affected local populations of this species (for instance, if this species does not have high population density near a city) or this species may have been missed in censuses of urban areas. These results indicate that phylogenetic grouping can be used to designate where conservation efforts should be focused, but that complete species inventories in urban and non‐urban regions across Africa are vital to determine what is known about urban tolerance in bats.

Our study highlights major gaps in the knowledge of bats in Africa, particularly their interaction with urbanization. The limited number of species for which both ecological and phylogenetic data were available indicates the need for further baseline research on African bats, especially in the Northern and Western regions of the continent. Moreover, there is limited information on presence/absence of bat species in urban areas, with remarkably few focused urban studies in Africa, which mostly focused on one region (Durban, South Africa; Schoeman, 2016; Schoeman & Waddington, 2011; Taylor et al., 1999). Unfortunately, published studies do not report the relevant levels of urbanization. These data are important to compare with levels of urbanization in Africa. Future studies should utilize data reported in a standardized manner (Wolf et al., 2022), controlling for the level of urbanization, surrounding micro‐habitats, and broadscale land use.

Our results show that resident urban bat species are pre‐adapted to successfully occupy urban environments. African bat species that are found in urban landscapes belong to particular phylogenetic groups and exhibit particular ecological traits including high mobility and flexible roosting strategies. Consequently, urbanization will probably reduce both functional and phylogenetic diversity of local bat faunas (McKinney, 2006; Morelli et al., 2016; Schoeman, 2016; Sol et al., 2017, 2020). This homogenization of bat diversity may lead to loss of key ecosystem services such as pest and disease control (Kalda et al., 2015; Kunz et al., 2011). Narrow space‐adapted species with high roost specificity are the most vulnerable to effects of urbanization in Africa. Therefore, conservation efforts and urban planning should focus on preserving suitable roost and foraging habitats for these species (McKinney, 2006; Morelli et al., 2016). Because bats of the African continent remain relatively understudied (Voigt & Kingston, 2016), more ecological and evolutionary data, particularly at fine geographic scales, are necessary to ensure that such conservation efforts are successful in urban landscapes across the continent.

## AUTHOR CONTRIBUTIONS

**Genevieve E. Marsden:** Data curation (equal); formal analysis (equal); methodology (equal); writing – original draft (equal). **Dalene Vosloo:** Project administration (equal); supervision (equal); writing – review and editing (equal). **M. Corrie Schoeman:** Conceptualization (equal); data curation (equal); methodology (equal); project administration (equal); resources (equal); supervision (equal); writing – review and editing (equal).

## ACKNOWLEDGEMENTS

This research was funded by the University of KwaZulu‐Natal and the South African National Research Foundation. We thank three anonymous reviewers who helped improve a previous version of the manuscript.

## CONFLICT OF INTEREST STATEMENT

The authors declare no conflict of interest.

## Data Availability

The full data set is available online at Dryad repository, DOI https://doi.org/10.5061/dryad.k3j9kd5b9.
